# The 3,4-Quinones of Estrone and Estradiol Are the Initiators of Cancer whereas Resveratrol and *N*-acetylcysteine Are the Preventers

**DOI:** 10.3390/ijms22158238

**Published:** 2021-07-30

**Authors:** Ercole Cavalieri, Eleanor Rogan

**Affiliations:** 1 Eppley Institute for Research in Cancer and Allied Diseases, University of Nebraska Medical Center, 986805 Nebraska Medical Center, Omaha, NE 68198-6805, USA; ecavalie@unmc.edu; 2 Department of Environmental, Agricultural and Occupational Health, University of Nebraska Medical Center, 984388 Nebraska Medical Center, Omaha, NE 68198-4388, USA

**Keywords:** cancer initiation, cancer prevention, estrogens, estrogen-DNA adducts, *N*-acetylcysteine, resveratrol

## Abstract

This article reviews evidence suggesting that a common mechanism of initiation leads to the development of many prevalent types of cancer. Endogenous estrogens, in the form of catechol estrogen-3,4-quinones, play a central role in this pathway of cancer initiation. The catechol estrogen-3,4-quinones react with specific purine bases in DNA to form depurinating estrogen-DNA adducts that generate apurinic sites. The apurinic sites can then lead to cancer-causing mutations. The process of cancer initiation has been demonstrated using results from test tube reactions, cultured mammalian cells, and human subjects. Increased amounts of estrogen-DNA adducts are found not only in people with several different types of cancer but also in women at high risk for breast cancer, indicating that the formation of adducts is on the pathway to cancer initiation. Two compounds, resveratrol, and *N*-acetylcysteine, are particularly good at preventing the formation of estrogen-DNA adducts in humans and are, thus, potential cancer-prevention compounds.

## 1. Introduction

Estrogens play a role in the development of multiple types of prevalent cancers. This relationship has been observed for more than a century. However, the role of estrogens has only been elucidated in recent years. Estrogen-induced cancers include breast, ovarian and endometrial cancer in women and prostate cancer in men. In postmenopausal women, a two-fold greater risk of breast cancer has been observed in women with higher levels of circulating estrogen than in women with lower levels [1]. Limited data have been compiled addressing this relationship in premenopausal women [1]. Prolonged exposure of women to elevated levels of endogenous estrogens has also been associated with an increased risk for breast cancer [2]. Data also support a relationship between levels of circulating estrogens and risk of ovarian or endometrial cancer in postmenopausal women [1].

The role of estrogens in the etiology of prostate cancer has been recognized for a shorter period of time. In 1995, Bosland et al. hypothesized that prostate cancer is initiated by estrogens and promoted by androgens [3]. More recently, epidemiologic evidence supports the role of estrogens in the etiology of prostate cancer [4]. For example, circulating estrogen levels in African-American men, who have the highest incidence of prostate cancer in the United States, are higher than those in Caucasian men [5]. On the other hand, Japanese men have a low incidence of prostate cancer and relatively low circulating estrogen levels compared to Dutch European men [6]. The ratio of total estradiol (E_2_) to total testosterone and total estrone (E_1_) to androstenedione was also found to be higher in Blacks than in other groups [7]. The enzyme aromatase catalyzes the conversion of androgens into estrogens. Aromatase activity has been demonstrated in the prostate, enabling the synthesis of estrogens from androgens in situ in the gland [8]. Interestingly, aromatase knock-out mice cannot produce E_2_ in the prostate, and they develop benign prostate hypertrophy rather than prostate cancer [9]. All of this information demonstrates the critical role of estrogens in the etiology of prostate cancer.

The oncogenic role of estrogens has been investigated in terms of hormonal, nonhormonal, and immunological responses. Chemical carcinogenesis, however, is the basis for the initiation of cancer. The chemicals that cause most human cancers are estrogens, and all of us, both men and women, form endogenous estrogens.

There is a common mechanism leading to the initiation of many prevalent types of cancer. Cancer can be initiated by increased formation of reactive estrogen metabolites called catechol estrogen-3,4-quinones. If estrogen metabolism becomes unbalanced and significant amounts of these quinones arise, depurinating estrogen-DNA adducts are primarily formed, leading to cancer-causing mutations [10,11,12,13,14].

A legitimate question can be raised about the relative importance of depurinating estrogen-DNA adducts compared with stable adducts. The depurinating adducts are quickly detached from the strand of DNA, leaving behind apurinic sites, whereas stable adducts remain in DNA unless removed by repair enzymes [13,14]. The essential role of depurinating DNA adducts, and the apurinic sites they generate during the initiation of cancer, was first demonstrated with adducts of three potent carcinogenic polycyclic aromatic hydrocarbons (PAH), namely: benzo[*a*]pyrene; 7,12-dimethylbenz[*a*]anthracene, and dibenzo[*a*,*l*]pyrene [13]. For all three PAH, the site of mutations in the H-*ras* oncogene in mouse skin tumors is dependent on the site of depurinating PAH-DNA adducts [15]. If the depurinating adducts are predominantly formed at adenines, the oncogenic H-*ras* mutations are at adenines. Similarly, the formation of depurinating adducts at guanines correlates with H-*ras* mutations at guanines in the tumors [15]. Similar results have been observed with depurinating estrogen-DNA adducts in both mouse skin [16] and rat mammary glands [17]. By contrast, there is no correlation between stable DNA adducts with oncogenic mutations. Thus, depurinating estrogen-DNA adducts play a critical role in the etiology of various prevalent types of cancer.

## 2. Initiation of Cancer

**Figure 1:**
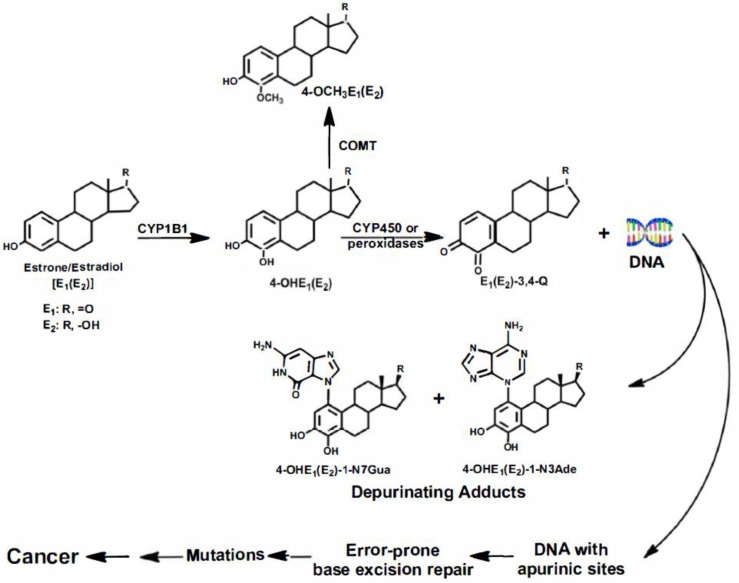
Pathway of cancer initiation by estrogens.

The pathway by which estrogens can cause cancer is shown in Figure 1. In this pathway, the estrogens estrone (E_1_) and estradiol (E_2_) are oxidized by cytochrome P450 (CYP)1B1 to the 4-catechol estrogens, 4-OHE_1_(E_2_) [18], which are further oxidized to the E_1_(E_2_)-3,4-quinones [E_1_(E_2_)-3,4-Q] by cytochrome P450 or peroxidases. Estrogen metabolism can be balanced or unbalanced in terms of the levels of catechol estrogen quinones formed. Normally, estrogen metabolism is balanced and low levels of E_1_(E_2_)-3,4-Q are formed, but when estrogen metabolism becomes unbalanced, higher levels of 4-OHE_1_(E_2_) and E_1_(E_2_)-3,4-Q are formed [11,12,13,14]; this has been demonstrated in the kidneys of Syrian golden hamsters [11], prostates of Noble rats [3,19], mammary glands of estrogen receptor knock-out (ERKO)/*Wnt-1* mice [20], and breasts of women [12].

The E_1_(E_2_)-3,4-Q overwhelmingly react with DNA at specific guanines and adenines to form the estrogen-DNA adducts 4-OHE_1_(E_2_)-1-N7Gua and 4-OHE_1_(E_2_)-1-N3Ade (Figure 1), which are released from DNA by depurination, leaving apurinic sites in the DNA [16,17]. Repair of the apurinic sites can lead to errors, resulting in mutations; this has been demonstrated in both mice and rats [16,17]. These genetically altered cells then undergo a series of changes resulting in the growth of malignant cells into detectable cancer. The development of estrogen-initiated cells into malignant tumors does not require the involvement of the estrogen receptor (ER), although the ER certainly plays a role in the promotion of cancer cells; the development of mammary tumors in 100% of female ERKO/Wnt-1 mice demonstrated this. These mice have no functional ER-α, and they have the *Wnt-1* transgene, which induces mammary tumors in all female mice [21,22]. Initially, it was thought that knocking out ER-α would demonstrate that the estrogen receptor is necessary for the development of mammary tumors, but the tumors developed in the absence of the receptor, disproving a critical role of the receptor in the development of mammary tumors [21,22]. However, these tumors are dependent on E_2_, and tumor development can be controlled by the dose of E_2_ given to ovariectomized females [23,24]. To further prove that ER-α does not play an essential role in the development of mammary tumors, it was found that tumors arise even in female ERKO/Wnt-1 mice treated with an antiestrogen known to block the receptor [25]. Nonetheless, mammary tumors develop in ERKO/Wnt-1 mice more slowly than in mice that have ER-α [22,23,24,25], suggesting a role of ER-α in tumor promotion.

The immortalized human breast epithelial MCF-10F cell line does not contain functional ER-α. Nonetheless, these cells can also be transformed by E_2_ into malignant cells that induce tumors when implanted into immunodeficient mice [26,27,28,29]. The transformation of MCF-10F cells into malignant cells can be increased or decreased by manipulating the levels and/or activity of estrogen-metabolizing enzymes such as CYP1B1 and catechol-*O*-methyltransferase (COMT) [30,31,32,33]. With more CYP1B1 activity, more E_1_(E_2_)-3,4-Q, higher levels of adducts, and more cell transformation occur. By contrast, with more COMT activity, less E_1_(E_2_)-3,4-Q, lower levels of adducts, and less cell transformation occur. MCF-10F cells are transformed by estrogens in the presence of antiestrogens, such as tamoxifen or ICI-182,780, demonstrating that the estrogen receptor does not play a role in the transformation [34]. These effects contribute to understanding how estrogens initiate cancer by metabolism into E_1_(E_2_)-3,4-Q, which react with DNA to generate specific mutations.

**Figure 2:**
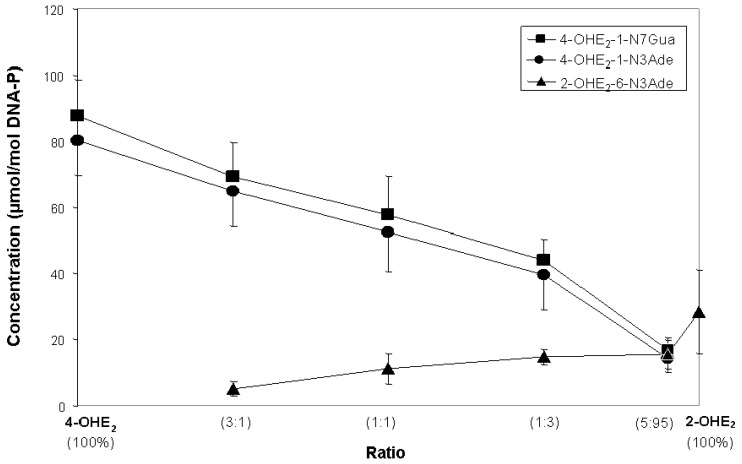
Depurinating adducts formed after 10 h (to allow complete depurination of 4-OHE_2_-1-N7Gua) by mixtures of lactoperoxidase-activated 4-OHE_2_ and 2-OHE_2_ reacted with DNA at different ratios. The level of stable adducts formed in the mixtures was less than 1% of the total adducts [37].

E_1_(E_2_)-3,4-Q are highly electrophilic compounds, seeking reaction with nucleophilic sites in the adenine (Ade) and guanine (Gua) bases in DNA [10,35,36]. The reaction of the electrophilic 3,4-keto groups with the N-7 of Gua and the N-3 of Ade occurs specifically at the nucleophilic C-1 position of the estrogen, resulting in the reduction of the vicinal keto groups to hydroxyl groups [13,14]; this causes the E_1_(E_2_)-3,4-Q to be far more reactive with DNA than the E_1_(E_2_)-2,3-Q, leading to many more depurinating DNA adducts formed with 4-OHE_1_(E_2_) than with 2-OHE_1_(E_2_). For example, in an in vitro reaction with DNA catalyzed by lactoperoxidase, a mixture of 95% 2-OHE_2_ and 5% 4-OHE_2_ is needed to form approximately similar amounts of the depurinating 2-OHE_2_ adducts as 4-OHE_2_ adducts (Figure 2) [37]. Similar results were obtained when mixtures of horseradish peroxidase-activated 2-OHE_2_ and 4-OHE_2_, or the quinones themselves, reacted with DNA [37]. In all of these reactions, the level of stable adducts formed was no more than approximately 1% of the total adducts.

It is not surprising, therefore, that the 4-OHE_1_(E_2_) are far more potent as carcinogens than the 2-OHE_1_(E_2_) [38,39,40]. The 4-OHE_1_(E_2_) induce kidney tumors in male Syrian golden hamsters, but the 2-OHE_1_(E_2_) do not [38,39]. Neonatal exposure of CD-1 mice to 4-OHE_2_ led to uterine adenocarcinomas, but 2-OHE_2_ had only borderline ability to induce the tumors [40]. These differences in carcinogenic potency cannot be attributed to redox cycling, which forms hydroxyl radicals, because 2-OHE_2_ and 4-OHE_2_ have identical redox potentials [41,42]. The formation of reactive oxygen species by oxidative stress has been shown to increase estrogen carcinogenesis [43]; this is considered by some to induce cancer-causing mutations, as evidenced by 8-oxo-dG [2]. However, this is not consistent with the greater potency of 4-OHE_1_(E_2_) compared with 2-OHE_1_(E_2_) [38,39,40]. Instead, the greater potency arises because E_1_(E_2_)-3,4-Q form depurinating DNA adducts more efficiently than E_1_(E_2_)-2,3-Q (Figure 2) [37].

Depurinating estrogen-DNA adducts can be detected in humans [13,14]. These adducts migrate out of cells and tissues, travel through the bloodstream, and are excreted in urine. By using ultraperformance liquid chromatography/tandem mass spectrometry (UPLC-MS/MS), the adducts 4-OHE_1_(E_2_)-1-N7Gua, 4-OHE_1_(E_2_)-1-N3Ade, and 2-OHE_1_(E_2_)-6-N3Ade can be detected in human urine and serum, although the level of the 2-OHE_1_(E_2_) adducts is generally much lower than the level of the 4-OHE_1_(E_2_) adducts [44,45,46,47,48,49,50,51]. However, the level of the adducts itself is not predictive of cancer risk since the level of estrogens in humans continuously varies. Instead, the molar ratio of depurinating estrogen-DNA adducts to estrogen metabolites and estrogen conjugates defines the balance of estrogen metabolism in a person and thus predicts that person’s risk for developing cancer [13,14,45]. This ratio is defined as follows:

$$\mathrm{ratio}=\left(\frac{{4-\mathrm{OHE}}_{1_{}}\left(E_{2}\right){-1-N3\mathrm{Ade}+4-\mathrm{OHE}}_{1_{}}\left(E_{2}\right)-1-N7\mathrm{Gua}}{4-\mathrm{catechol}\;\mathrm{estrogens}+4-\mathrm{catechal}\;\mathrm{estrogen}\;\mathrm{conjugates}}+\frac{{2-\mathrm{OHE}}_{1_{}}\left(E_{2}\right)-6-N3\mathrm{Ade}}{2-\mathrm{catechol}\;\mathrm{estrogens}+2-\mathrm{catechal}\;\mathrm{estrogen}\;\mathrm{conjugates}}\right)\times \;1000$$

**Figure 3:**
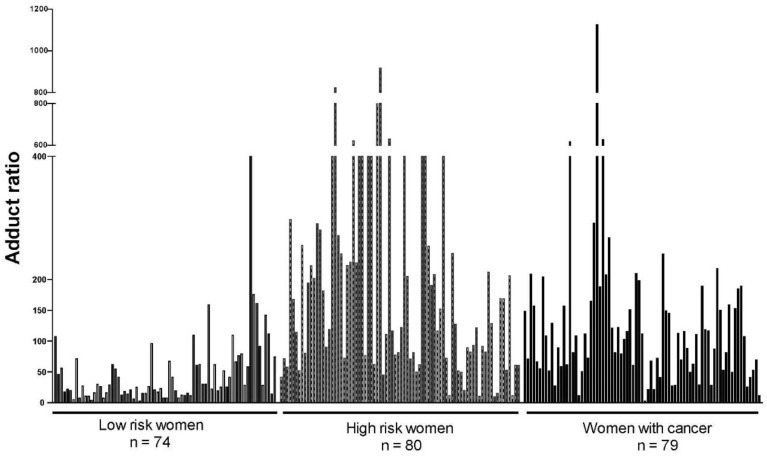
Ratios of depurinating estrogen-DNA adducts to estrogen metabolites and conjugates in serum of women at low risk, women at high risk and women diagnosed with breast cancer (*p* < 0.0001) [46].

The ratio of depurinating estrogen-DNA adducts to estrogen metabolites and conjugates has been analyzed extensively in women diagnosed with breast cancer compared with women at normal or high risk of developing the disease (Figure 3) [44,45,46]. This ratio has no units because they cancel out between the numerator and denominator. The ratio in women at normal risk of developing breast cancer was significantly lower than the ratio in women at high risk or diagnosed with breast cancer (*p* < 0.001) in three different studies [44,45,46]. Similar estrogen-DNA adduct ratios are found in postmenopausal women and premenopausal women [46], establishing that the balance of estrogen metabolism does not depend on the level of circulating estrogens in a person. Instead, the balance of estrogen metabolism depends on a person’s enzymological makeup and can be affected by environmental factors that alter the balance of enzyme activities.

In the initial study, estrogen-DNA adduct ratios in urine samples from 46 normal-risk women, 12 high-risk women, and 17 women with breast cancer were compared, and the average ratio of the normal-risk women was significantly lower than the average ratios of the high-risk and women with breast cancer (both *p* < 0.001) [44]. In the second study, groups of 40 women at normal risk, high risk, or diagnosed with breast cancer were compared; again, the average ratio of the normal-risk women was significantly lower than those of the other two groups (both *p* < 0.001) [45]. In the third study, estrogen-DNA adduct ratios in serum from 74 women at normal risk, 80 women at high risk, and 79 women diagnosed with breast cancer were compared; the average ratio in the normal-risk women was again significantly lower than those in the high-risk and breast cancer groups (both *p* < 0.0001, Figure 3) [46]. By contrast, the groups of women at high risk or diagnosed with breast cancer have similarly high average ratios (Figure 3) [44,45,46]. The critical observation from these studies is that women at high risk of developing breast cancer have a high ratio of adducts to metabolites and conjugates. This finding indicates that the formation of depurinating estrogen-DNA adducts begins before breast cancer develops and suggests that the formation of these adducts is a critical event in the initiation of cancer.

The three studies of women described above were conducted primarily with Caucasian women. An additional study was conducted with African-American women, but in this study, the women were divided into only two groups: those diagnosed with breast cancer and those who had not been diagnosed with breast cancer [47]. Once again, a significant difference was observed in the estrogen-DNA adduct ratios in the two groups of women. The women not diagnosed with breast cancer had an average ratio of 38.5, whereas those diagnosed with breast cancer had an average ratio of 92.4 (*p* < 0.001) [47]. These results support the role of estrogen-DNA adducts in the initiation of breast cancer.

The ratio of depurinating estrogen-DNA adducts to estrogen metabolites and conjugates are also found to be significantly higher in women with ovarian (*p* < 0.0001) or thyroid (*p* < 0.0001) cancer [48,49]. The study of women with and without ovarian cancer included urine samples for analysis of estrogen metabolites, conjugates and depurinating DNA adducts, as well as saliva samples for purification of DNA and analysis of single nucleotide polymorphisms (SNPs) in several genes for the enzymes involved in estrogen metabolism, CYP1B1 and COMT [49]. The selected SNPs resulted in a more active CYP1B1, which increased the formation of E_1_(E_2_)-3,4-Q, and a less active COMT, which decreased methylation of 4-OHE_1_(E_2_), thereby increasing the amount of E_1_(E_2_)-3,4-Q formed. Both of these SNPs thus resulted in more E_1_(E_2_)-3,4-Q available to react with DNA. When women had one or two copies of the SNP for a more active CYP1B1 plus two copies of the SNP for a less active COMT, they were three times more likely to have ovarian cancer, and had approximately twice the ratio of estrogen-DNA adducts to estrogen metabolites and conjugates as women without the SNPs. When the women had two copies of both the CYP1B1 and COMT SNPs, they were six times more likely to have the disease and had even higher estrogen-DNA adduct ratios [49]. Similar to the high ratios of estrogen-DNA adducts to estrogen metabolites and conjugates in women at high risk for breast cancer, these results suggest that the formation of estrogen-DNA adducts is a precursor to the development of cancer.

As we know, estrogens are present in both men and women. Therefore, estrogen metabolites, estrogen conjugates, and estrogen-DNA adducts can be analyzed in men and women, and the ratio of adducts to metabolites and conjugates can be determined. In fact, the first study of depurinating estrogen-DNA adducts in humans was conducted in men with and without prostate cancer [52]. As noted previously, Bosland et al. hypothesized that prostate cancer is initiated by estrogens and promoted by androgens [3]. In agreement with this hypothesis, it was found that men with prostate cancer have significantly higher ratios of adducts to metabolites and conjugates (*p* < 0.001) than healthy men not diagnosed with cancer [50]. The same observation was made in men with and without non-Hodgkin lymphoma (*p* < 0.0007) [51].

In summary, these results in both men and women strongly suggest that the formation of depurinating estrogen-DNA adducts is a critical event in the initiation of cancer.

## 3. Prevention of Cancer by Inhibition of Estrogen-DNA Adduct Formation

Estrogens need to be metabolized to the reactive catechol estrogen-3,4-quinones to be carcinogenic. Therefore, it should be possible to prevent cancer by inhibiting this metabolic process and/or blocking reaction of the quinones with DNA. Two compounds are particularly good at blocking the metabolism of estrogens to quinones and/or scavenging the reactive quinones, they are: resveratrol and *N*-acetylcysteine (NAC) [13,14,30,31,32,53]. Cysteine is equally effective but it cannot be used because of its toxicity in humans. Resveratrol is a natural product found in grape skins, red wine, and peanuts, and it has various biological actions. Resveratrol is found to have several therapeutic effects; it is known to be anti-inflammatory, antioxidant, antihyperlipidemic, and anticarcinogenic, possessing immune-modulating, cardioprotective, hepatoprotective, and neuroprotective properties [54]. Among these effects are specific abilities to reduce catechol estrogen semiquinones back to catechol estrogens [30,53] and induce the estrogen-protective enzyme quinone reductase [30,31,32,53]. Resveratrol also modulates CYP1B1, thereby reducing its activity and thus the formation of 4-OHE_1_(E_2_). NAC has somewhat different properties. It, too, can reduce estrogen semiquinones back to catechol estrogens, but its primary effect is to react with quinones to form conjugates [30,31,32,53], thus preventing the formation of estrogen-DNA adducts.

**Figure 4:**
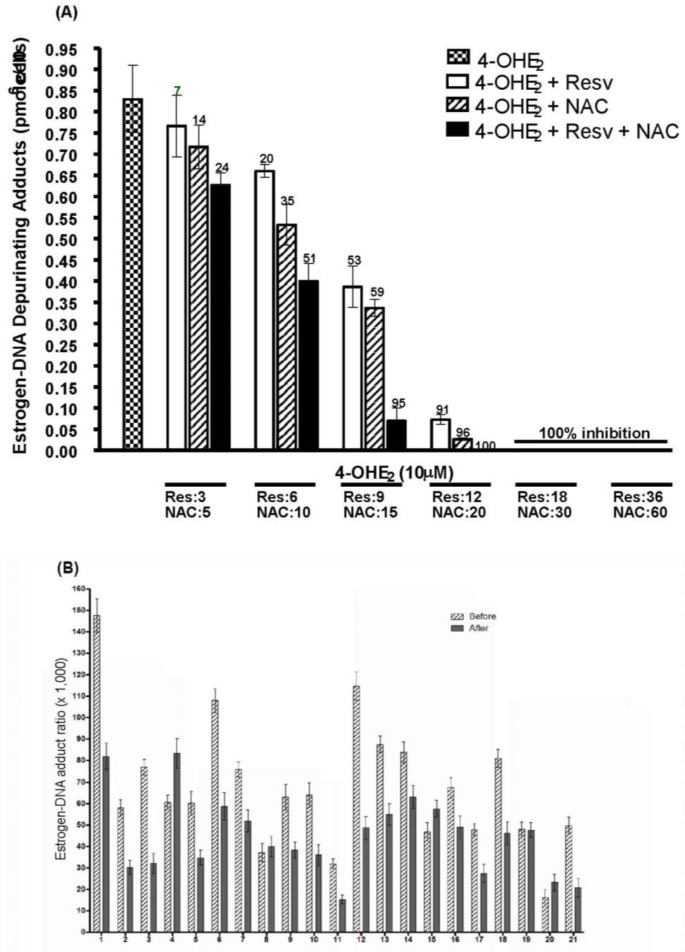
Inhibition of estrogen-DNA adduct formation by *N*-acetylcysteine and resveratrol in (**A**) MCF-10F cells treated with 4-OHE_2_ [30]; and (**B**) urine from healthy women before and after 3 months of daily ingestion of the two compounds [56].

Both resveratrol and NAC have been shown to inhibit the formation of depurinating estrogen-DNA adducts in cultured mammalian cells [30,31,32]. Resveratrol was found to inhibit the malignant transformation of the human MCF-10F breast epithelial cell line [31]. NAC was found to inhibit the malignant transformation of both MCF-10F and immortalized mouse mammary cells [33,55]. The two compounds work together additively to reduce the formation of depurinating estrogen-DNA adducts in MCF-10F cells treated with 4-OHE_2_ (Figure 4A) [30]. These results lay the foundation for investigating the ability of resveratrol and NAC to reduce estrogen-DNA adduct formation in humans as an approach to cancer prevention.

In order to consider resveratrol and/or NAC as potential cancer-preventing compounds, it is necessary to demonstrate their ability to reduce the formation of estrogen-DNA adducts in humans. Therefore, a study was conducted to test the two compounds’ ability to affect adduct formation in normal healthy women. A total of 21 women provided a urine sample before and after a 3-month treatment regimen that included taking resveratrol and NAC daily for 3 months. The urine samples were analyzed for estrogen metabolites, conjugates, and depurinating DNA adducts. In these women, the ratio of estrogen-DNA adducts to estrogen metabolites and conjugates was reduced by a statistically significant amount (*p* = 0.03, Figure 4B) during the three-month study period [56]. These results suggest that ingestion of NAC and resveratrol may constitute a safe, low-cost, effective measure to prevent breast cancer and other types of cancer.

## 4. Conclusions

Estrogens initiate cancer through metabolism to catechol estrogen-3,4-quinones. The quinones react with DNA to primarily form the depurinating DNA adducts 4-OHE_1_(E_2_)-1-N7Gua and 4-OHE_1_(E_2_)-1-N3Ade. The resulting apurinic sites in specific locations in DNA generate mutations that can initiate cancer; this has been shown in laboratory animals and human beings. Two compounds, resveratrol and NAC, are potential cancer-preventive compounds that can reduce the formation of estrogen-DNA adducts in people, thereby blocking the initiation of estrogen-induced cancer.

The two major mechanisms of estrogen-induced cancer are ER-dependent and ER-independent mechanisms. A variety of evidence has been cited to support an ER-dependent mechanism. Working through ER-α, estrogens increase the rate of cell proliferation, which decreases opportunities for cells to repair mutations; in turn, this may increase the risk for malignant transformation [57]. Additionally, the ability of anti-estrogens tamoxifen and raloxifene to prevent breast cancer in women also supports an ER-dependent mechanism [58,59,60].

Other evidence points to an ER-independent mechanism. The development of estrogen-dependent mammary tumors in ERKO/Wnt-1 mice, even in the presence of an anti-estrogen, constitutes some of the strongest evidence for an ER-independent mechanism of cancer initiation by estrogens. The observation of high ratios of depurinating estrogen-DNA adducts to estrogen metabolites and conjugates in women at high risk for breast cancer, as well as women with breast cancer [45,46,47], is consistent with an ER-independent mechanism of initiation. In addition, the presence of SNPs in CYP1B1 and COMT that increase both the formation of depurinating estrogen-DNA adducts and the likelihood of ovarian cancer (six-fold) [50] supports an ER-independent mechanism of cancer initiation by estrogens.

Of the estimated number of people in the United States who died of cancer in 2020 (606,520) [61], 140,000 were smokers. Cancer would have been preventable for approximately 466,000 people by ingestion of resveratrol and NAC; only the smokers would have died because they produce different carcinogens while smoking. If people cease their smoking habits, cancer may become a rare disease for human beings.

## Data Availability

To obtain the data used in the results reported in this article contact Eleanor Rogan.
